# Local environmental and meteorological conditions influencing the invasive mosquito *Ae*. *albopictus* and arbovirus transmission risk in New York City

**DOI:** 10.1371/journal.pntd.0005828

**Published:** 2017-08-23

**Authors:** Eliza Little, Waheed Bajwa, Jeffrey Shaman

**Affiliations:** 1 Environmental Health Sciences, Mailman School of Public Health, Columbia University, New York, New York, United States of America; 2 Office of Vector Surveillance and Control, New York City Department of Health and Mental Hygiene, New York, New York, United States of America; Centers for Disease Control and Prevention, UNITED STATES

## Abstract

*Ae*. *albopictus*, an invasive mosquito vector now endemic to much of the northeastern US, is a significant public health threat both as a nuisance biter and vector of disease (e.g. chikungunya virus). Here, we aim to quantify the relationships between local environmental and meteorological conditions and the abundance of *Ae*. *albopictus* mosquitoes in New York City. Using statistical modeling, we create a fine-scale spatially explicit risk map of *Ae*. *albopictus* abundance and validate the accuracy of spatiotemporal model predictions using observational data from 2016. We find that the spatial variability of annual *Ae*. *albopictus* abundance is greater than its temporal variability in New York City but that both local environmental and meteorological conditions are associated with *Ae*. *albopictus* numbers. Specifically, key land use characteristics, including open spaces, residential areas, and vacant lots, and spring and early summer meteorological conditions are associated with annual *Ae*. *albopictus* abundance. In addition, we investigate the distribution of imported chikungunya cases during 2014 and use these data to delineate areas with the highest rates of arboviral importation. We show that the spatial distribution of imported arboviral cases has been mostly discordant with mosquito production and thus, to date, has provided a check on local arboviral transmission in New York City. We do, however, find concordant areas where high *Ae*. *albopictus* abundance and chikungunya importation co-occur. Public health and vector control officials should prioritize control efforts to these areas and thus more cost effectively reduce the risk of local arboviral transmission. The methods applied here can be used to monitor and identify areas of risk for other imported vector-borne diseases.

## Author summary

This paper examines the ecological underpinnings of the invasive mosquito *Ae*. *albopictus* and the associated risk of arboviral transmission in New York City. We aim to quantify the relationships between local environmental and meteorological conditions and *Ae*. *albopctus* abundance. Further, we explicitly determine risk of local arbovirus disease transmission by *Ae*. *albopictus* by overlaying imported chikungunya cases from the epidemic year of 2014. Our overarching objective is to determine the extent of *Ae*. *albopictus* infestation and the distribution of viremic human hosts to predict risk of localized chikungunya outbreaks in New York City, and use these predictions to focus vector control and community education interventions to localities at greatest risk. We develop a model incorporating both local environmental and meteorological conditions to predict *Ae*. *albopictus* populations at fine spatial scale. We find that peak imported chikungunya cases and *Ae*. *albopictus* populations are temporally synchronous but primarily spatially asynchronous. The areas that do have high arboviral importation and *Ae*. *albopictus* populations should be prioritized for vector control and education interventions.

## Introduction

*Aedes albopictus* Skuse 1984, also known as the Asian tiger mosquito, is an invasive mosquito of growing consequence and concern especially for temperate areas [1, 2]. Originating from Southeast Asia, this mosquito has expanded its range globally over the past three decades [3]. Its invasiveness is linked to its ability to exploit a range of container habitats, to lay desiccation resistant eggs that can survive without water for up to a year, and to oviposit eggs that hatch in installments [3]. In North America it was first observed in Texas in 1985 and its spread to the northeastern US was linked to the highway network [4]. To date there are over 500 counties in 34 states as well as the District of Columbia where *Ae*. *albopictus* has been reported [5, 6].

In the last two decades, the Americas have witnessed the emergence of a number of epidemic arboviruses of public health significance: Beginning in the 1990s the resurgence and spread of dengue (DENV), in 1999 the arrival of West Nile virus (WNV), and in 2013 the explosive spread of chikungunya (CHIKV). In the past year, the western hemisphere has experienced yet another arbovirus, Zika (ZIKV). These diseases incur significant costs to local economies and health care systems. Acute symptoms are typically not life-threatening; however, chronic conditions associated with these arboviruses are serious and in the case of the link between ZIKV and congenital microcephaly, particularly devastating.

*Ae*. *aegypti* readily transmits arboviruses to humans due to its anthropophilic biting tendencies; this vector lives in close proximity to humans and almost exclusively bite people. In contrast, *Ae*. *albopictus* is often considered a secondary vector of human arboviruses, because it inhabits a wider range of environments, including suburban and rural, and bites a wider variety of hosts, including birds [7]. These factors mitigate its transmission potential to humans. The principal argument cited for its secondary role is that in areas where it is present and *Ae*. *aegypti* is absent outbreaks are limited [8, 9]. However, the role of *Ae*. *albopictus* as a vector has not been fully elucidated across much of its range, particularly in places where it has recently been introduced, such as Europe (1979) and North America (1985) [5, 6, 10]. Its role may be secondary to *Ae*. *aegypti*; it may still be evolving; it may be the primary vector in more suburban and rural areas; it may be an important vector bridging sylvatic and urban cycles; or it may have an important role maintaining viruses between epidemics [11, 12]. It is also possible that *Ae*. *albopictus* behaves differently depending on its environment, whether urban, suburban or rural [13].

In its native range, *Ae*. *albopictus* mainly occurs in vegetated and rural habitats, especially where it co-occurs with *Ae*. *aegypti* [12]. However in areas where *Ae*. *aegypti* is absent, *Ae*. *albopictus* pullulates in urban areas [14]. As its range increases, *Ae*. *albopictus* appears to be more closely associated with humans [15]. Additionally, there is growing evidence that in human-dominated landscapes, *Ae*. *albopictus* favors humans, with 68–100% of blood meals taken from humans across nine studies recently reviewed [16]. Finally, its importance as a nuisance-biter further underscores its predilection for human blood when it is available [17, 18].

In temperate areas, where *Ae*. *aegypti* populations are limited by freezing temperatures, *Ae*. *albopictus* is the only endemic vector of DENV, YFV, CHIKV, and ZIKV. While temperate outbreaks occur they tend to be mild due to: the seasonality of mosquito populations limiting outbreaks at the onset of cold temperatures; sanitation services and piped water that reduce breeding habitats; infrastructural barriers, including screens and air conditioning that limit vector-host contact; and surveillance systems and other vector control resources that limit transmission if a local outbreak should arise [6, 19]. However temperate outbreaks do occur and may even be increasing in frequency.

*Ae*. *albopictus* has been implicated in the local spread of arboviruses in Asia, Europe, and the US. *Ae*. *albopictus* was responsible for frequent and widespread DENV epidemics in Japan during WWII, a DENV outbreak in Hawaii during 1943 [11], and DENV transmission in tropical regions of Asia until its displacement by *Ae*. *aegypti* in the 1950s [11]. More recently, *Ae*. *albopictus* was identified as the vector of the 2005–2007 CHIKV epidemic outbreak on La Reunion and in some of the outbreaks in India during the same time period [20]. In Europe, the first CHIKV outbreak occurred in Ravenna, Italy during 2007 with over 200 cases traced back to a single infected returning traveler and spread by established local populations of *Ae*. *albopictus* [21]. Subsequently, in France, local transmission of CHIKV by *Ae*. *albopictus* occurred in 2010 [22] and again in 2014 [23]. *Ae*. *albopictus* was also responsible for outbreaks of DENV in Asia: during 2001 and 2010 in China [24, 25] and 2014 in Japan [26]. In the US, *Ae*. *albopictus* mosquitoes caused a DENV outbreak in Hawaii during 2001 and a single locally acquired case in New York was attributed to *Ae*. *albopictus* in 2013 [27]. The recent invasion of *Ae*. *albopictus* in Gabon in 2007 was linked to the emergence of DENV, CHIKV, and ZIKV there [28].

In addition to the many arboviral outbreaks linked to *Ae*. *albopictus*, there are numerous other arboviruses that *Ae*. *albopictus* is known to carry, although its vectorial role remains largely un-described. Regardless, its broad viral susceptibility suggests that it may be implicated as an important, if not primary, vector in the transmission of other arboviruses now and in the future [11].

Even in the absence of disease transmission, infestation with *Ae*. *albopictus* may accrue negative health outcomes. In the eastern US, it has become the most common nuisance mosquito, aggressively biting humans during the day—so much so that it is a leading deterrent of outdoor recreation in cities [11, 17, 18, 29].

New York City (NYC) is a hub for international travel, which increases the chance of arbovirus introduction into local *Ae*. *albopictus* populations. There have been many arbovirus cases imported into New York: during 2014, 803 imported CHIKV cases representing 29% of all US imported cases, and during 2016, 1001 ZIKV cases representing 21% of all US imported cases [30, 31]. True importation rates are likely higher given the asymptomatic rates of these diseases (25% for CHIKV and 80% for ZIKV [32, 33]). Given this high rate of importation, it is logical to investigate whether the conditions necessary for local arbovirus transmission—the mosquito vector, the virus, and the ecological and epidemiological conditions suitable for transmission—co-occur in NYC. Our aims for this study are to identify the factors affecting *Ae*. *albopictus* abundance and the importation of arbovirus cases, and to use these findings to develop spatial-temporal risk maps that can inform vector control strategies.

## Materials and methods

### Entomological data

The New York Department of Health and Mental Hygiene’s (NYC DOHMH) Office of Vector Surveillance and Control has 52 permanent mosquito surveillance sites spanning the five boroughs of NYC (S1 Fig). These 52 sites were established in 1999 after the introduction of WNV to NYC, and remained in operation each season from June 1st to October 31st. The trap locations and trap types deployed (gravid and light traps) are specifically targeted to collect WNV vectors (i.e. *Culex* mosquitoes). While not as effective as BG Sentinel traps for detecting the presence (especially low numbers) of *Ae*. *albopictus* [34, 35], these traps have been used to determine *Ae*. *albopictus* distribution and abundance [15, 36]. A recent study found BG and CDC light traps baited with dry ice like those in NYC to have equivalent *Ae*. *albopictus* trapping efficiency [36]. Weekly data from the light and gravid traps were combined as has been done previously to reduce bias and increase the power of analysis [15].

### Meteorological and local environmental conditions

Our modeling approach exploits links between meteorological and local environmental factors and *Ae*. *albopictus* populations in the northeastern US (see Supporting Information). To measure temporal differences in meteorological factors in NYC we used the North American Land Data Assimilation System (NLDAS) dataset, a combined NASA/NOAA product, which provides gridded estimates of near-surface meteorological conditions at 13 km x 13 km spatial resolution [37]. Hourly estimates of precipitation measured in millimeters per hour, temperature measured in Kelvin 2-m above ground, and specific humidity measured in kilograms per kilograms 2-m above ground were used to calculate monthly averages for the years 2006–2016.

To measure fine-scale spatial differences in the urban environment we used 3 foot spatial resolution land cover data [38]. This land cover dataset defines 7 land cover classes (trees, grass, bare, building, road, other paved, and water). We further calculated the Shannon diversity index (SDI) at the same 3 foot spatial resolution, which provides an estimate of environmental heterogeneity accounting for both the total proportional area of each land cover class (abundance) as well as the number of land cover classes present (evenness):

$$\begin{array}{c} SDI=\sum_{i=1}^{R}p_{i}ln\left(p_{i}\right) \end{array}$$ (1)

where the proportion of land cover class *i* relative to the total number of classes (*p*_*i*_) is multiplied by the natural logarithm of this proportion (*lnp*_*i*_), summed across classes, and multiplied by −1.

To determine the area covered by one or two family residential buildings, open spaces, and vacant lots we used data from PLUTO, a geographically registered dataset created by the Department of City Planning at the tax lot level for the city of New York [39]. We created raster grids of the PLUTO data at the same spatial resolution as the land cover classes.

We calculated the proportion of each of the 11 environmental variables (7 land cover, SDI, and 3 PLUTO) within 200m of every pixel in the mapped domain representing NYC. Because *Ae*. *albopictus* has a flight range under 200m [40], each pixel (which supplies an accounting of each of the 11 environmental variables within the 200m radius) provides a synopsis of the environmental conditions *Ae*. *albopictus* would be exposed to if present at that location in NYC. Next, we standardized these values by subtracting the mean and dividing by the standard deviation across the whole domain [41]. We extracted the standardized values at each of the 52 permanent trap locations to estimate local environmental conditions in order to model annual *Ae*. *albopictus* abundance.

#### Modeling

We employed an ensemble modeling approach, here defined as the formal weighted averaging of simulations from multiple models. Ensemble modeling was carried out in order to improve overall model fit and reconcile competing predictions. To select the set of models used for ensemble modeling we first selected only those models for which all explanatory variables were significant with 95% confidence. Models were then ranked by goodness-of-fit estimated using a second order Akaike Information Criterion (AICc), which more accurately takes into consideration the number of parameters in the model and provides better comparison across models with different numbers of parameters. For each model we calculated the Akaike weight, a relative measure of the model plausibility compared to the best fitting model (the model with the lowest AICc) given the data. The ensemble set is determined as the smallest subset of models whose Akaike weights sum to 0.95. This ensemble set was used to make parameter inferences and to calculate model averaged predictions with unconditional confidence intervals (for more information see [42]).

To assess the temporal influence of meteorological conditions we used a generalized linear negative binomial model (link = log) with trap location as a random effect to assess the influence of monthly meteorological conditions on the observed annual trap count of *Ae*. *albopictus*. Models using combinations of 4 monthly meteorological conditions restricted to January through August of each year were tested as predictors of annual abundance. In this lagged model form, the influence of monthly meteorological variables on annual abundance within a given year were considered but across years were not.

To understand the spatial influence of local environmental conditions we used a generalized linear negative binomial model (link = log) with year as a random effect to assess the influence of local environmental characteristics on observed annual abundance of *Ae*. *albopictus* for each trap location. We tested all possible combinations of the 11 explanatory environmental variables.

Parameters from the temporal and spatial model ensembles of high importance were retained for use in a spatiotemporal model. Parameter importance was calculated by tallying the Akaike model weights for each model in the ensemble for which the parameter was included. Thus a greater score indicates greater parameter importance compared to other parameters tested. Parameters of high importance were determined as those with a score greater than 0.5.

For the spatiotemporal model form we used a generalized linear negative binomial model without random effects, which assumes that inclusion of both environmental and meteorological factors explains the latent spatiotemporal variability characterized by the random effects in the separate temporal and spatial models. For a schematic representation of the methodological steps taken see S2 Fig.

We used the package glmmADMB to fit mixed effect models [43, 44] and MuMIn for model averaging [45]. All analyses were run in R [46].

#### Validation, prediction and mapping

To validate model performance, we used leave-one-out temporal cross validation (LOOCTV). Each year of data (2006–2016) was iteratively omitted from the analysis and the accuracy of the compiled set of predictions from the LOOTCV models was then compared to the predictions based on the full record. In addition, weighted average predictions were generated for 2016 using the spatiotemporal ensemble model. The accuracy of these predictions was evaluated using the measured annual abundance of *Ae*. *albopictus* at the 52 trapping sites during 2016. To the best of our knowledge this study represents the first fine scale prediction effort of *Ae*. *albopictus* abundance that includes validation based not just on LOOCTV but also out-of-sample data.

To map the empirical relationships we used the raster surface layers depicting the standardized departures for each of the environmental variables as described earlier (see materials and methods subsection meteorological and local environmental conditions). Raster surface layers of meteorological variables were created through interpolation based on the spatial locations of the NLDAS grid centroids. We then multiplied each corresponding surface raster layer with the coefficient estimated through ensemble spatiotemporal modeling. The resulting map provides a detailed spatial prediction of the abundance of *Ae*. *albopictus* throughout NYC given the parameters investigated.

#### Distribution of imported CHIKV cases in 2014

Both the mosquito vector and arbovirus are needed to support local transmission of disease. To determine where the second factor, the arbovirus, is likely to be introduced, we investigated imported CHIKV cases in NYC during 2014. Imported CHIKV cases in NYC were provided at the zipcode level by the Zoonotic, Influenza and Vector-borne Disease Unit of the NYC DOHMH for each month of 2014. 599 cases of CHIKV were reported between May and December 2014 across 124 zipcodes. Again, these numbers are likely an underestimate as 25–50% of infections are asymptomatic [33, 47–49]. We calculated the standardized ratio for the number of CHIKV cases reported in NYC during 2014 as the number of observed cases divided by the number of expected CHIKV cases for each zipcode (*O*_*i*_/*E*_*i*_). The expected number of CHIKV cases (*E*_*i*_) was calculated by multiplying the population for each zipcode (Pi) by the ratio of observed cases to the population across all zipcodes (*O*_+_/*P*_+_). By taking into account the population estimate for each zipcode we are able to compare across zipcodes with differing underlying populations and more accurately assess risk of CHIKV importation. Further, we used a spatiotemporal Poisson probability model in the program SatScan [50] to detect hot spots of CHIKV cases in time and space during the 2014 epidemic in NYC.

#### Risk of autochthonous disease spread

We calculated the mean predicted value of *Ae*. *albopictus* abundance for 2016 for each zipcode in NYC. The zipcode scale is the spatial unit for vector control efforts in NYC and the scale at which the arbovirus data are available from the NYC DOHMH. We categorized predicted zipcode level values of *Ae*. *albopictus* abundance and imported CHIKV cases into four categories by quartile. To identify three levels of risk (zipcodes of concordant high *Ae*. *albopictus* abundance and high risk of arbovirus importation) we determined *Ae*. *albopictus* and imported CHIKV cases counts above respective first quartile values, above respective mean values, and above respective third quartile values.

## Results

### Entomological data

**Fig 1:**
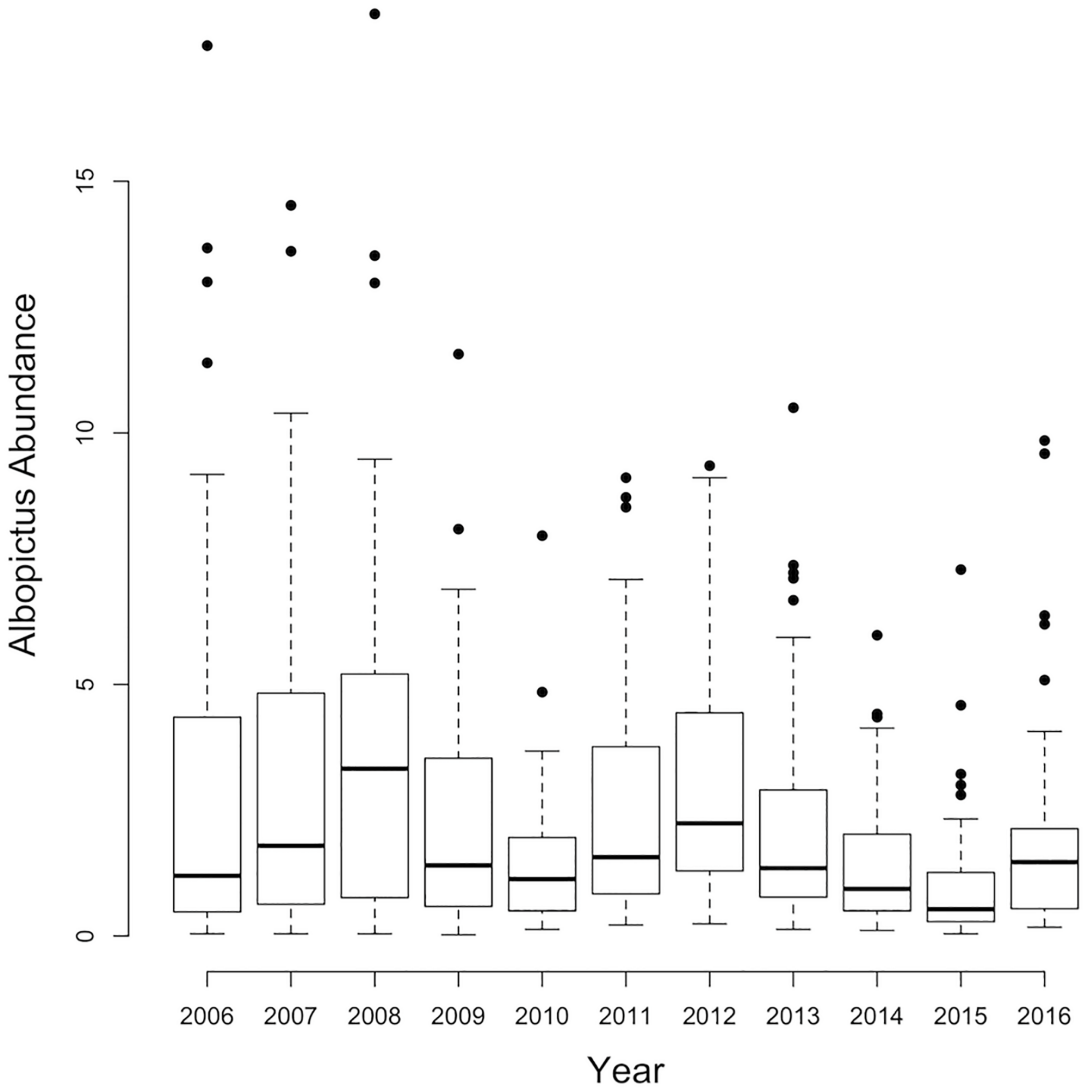
*Ae*. *albopictus* abundance. Box and whisker plots showing variability in annual *Ae*. *albopictus* abundance across 52 permanent trap locations in New York City 2006–2016. The box delimits the interquartile range, the whiskers extend 1.5 times the interquartile range, and the dots are outliers.

The surveillance data provide a record of the invasion and establishment of *Ae*. *albopictus* in NYC. This mosquito was first trapped in the Bronx during 2000, between 2000 and 2005 was caught in increasing trap numbers across the city, and between 2006 and 2016 was caught in over 96% of traps. We thus restricted our analysis to the period after invasion from 2006 to 2016. Between 2006 and 2016, 61,977 *Ae*. *albopictus* mosquitoes were caught in gravid and light traps across the 52 permanent trap locations. In 2016, BG Sentinel traps were added to the 52 permanent trap locations, trap counts from these BG Sentinel traps and the CDC light traps were significantly correlated (r = 0.21; p< .001). The annual numbers of traps collecting *Ae*. *albopictus* (traps positive), the total *Ae*. *albopictus* mosquitoes caught in gravid and light traps, and the abundance (calculated as the number caught per trap location divided by the 23 weeks of surveillance) for gravid, light, and both trap types together are shown in Table 1 and Fig 1.

**Table 1:** Overview of entomologic data showing the number of traps collecting *Ae*. *albopictus* (traps positive), the total mosquitoes caught in gravid and light traps, and the abundance (calculated as the number caught per trap location divided by the 23 weeks of surveillance) for gravid, light, and both trap types together.

Year	Traps Positive (%)	Total Gravid	Mean (SD) Gravid Abundance	Total Light	Mean (SD) Light Abundance	Mean (SD) Abundance
2006	50 (96%)	2386	2.07 (2.64)	4883	4.25 (5.79)	3.16 (4.04)
2007	50 (96%)	3107	2.70 (315)	3977	3.46 (4.38)	3.08 (3.37)
2008	51 (98%)	4332	3.70 (3.38)	4373	3.73 (4.44)	3.71 (3.74)
2009	52 (100%)	2456	2.05 (2.35)	3074	2.57 (2.88)	2.31 (2.43)
2010	52 (100%)	1427	1.19 (1.08)	2038	1.70 (2.13)	1.15 (1.41)
2011	52 (100%)	2687	2.25 (2.57)	3610	3.02 (3.10)	2.63 (2.43)
2012	52 (100%)	3679	3.08 (2.96)	4075	3.41 (3.13)	3.24 (2.67)
2013	52 (100%)	2255	1.89 (1.93)	3149	2.63 (2.99)	2.26 (2.27)
2014	52 (100%)	1526	1.28 (1.62)	1914	1.60 (1.32)	1.43 (1.30)
2015	52 (100%)	1190	0.99 (1.31)	1202	1.01 (1.30)	1.00 (1.28)
2016	52 (100%)	1914	1.60 (1.92)	2723	2.28 (2.84)	1.94 (2.12)

### Spatiotemporal modeling

**Fig 2:**
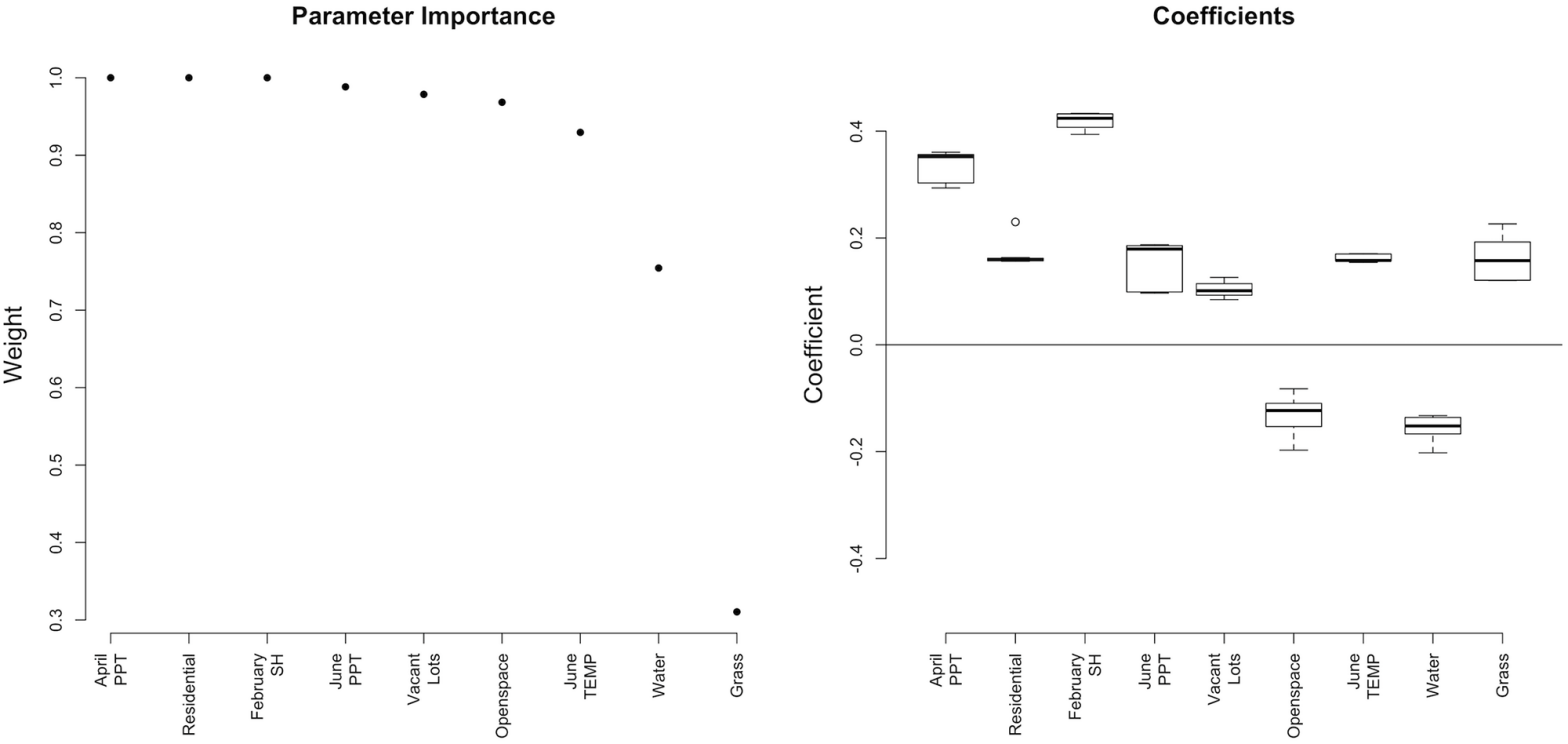
Spatiotemporal modeling results. Ranked order of predictor variable importance and coefficients for the ensemble model inference set.

The subset of important parameters from the spatial and temporal modeling efforts include February specific humidity, April precipitation, June temperature, and June precipitation, as well as the extent of residential buildings, open spaces, vacant lots, water, and grass. With these nine variables we fit generalized linear negative binomial models using all combinations of these variables. Of those tested, 137 were significant and 10 were included in the ensemble model set (Table 2; Fig 2).

**Table 2:** Spatiotemporal ensemble model set ranked by AICc.

Model	AICc	Weight	Open Space	Residential	Vacant Lots	Water	Grass	April PPT	Feb. SH	June PPT	June TEMP
1	1947.05	0.42	-0.15	0.16	0.08		0.15	0.34	0.40	0.18	0.16
2	1947.22	0.38	-0.08	0.16	0.10	-0.14		0.35	0.41	0.18	0.16
3	1951.92	0.04		0.23	0.12	-0.18		0.35	0.41	0.18	0.15
4	1951.96	0.04	-0.11	0.16	0.11			0.34	0.41	0.17	0.16
5	1953.16	0.02	-0.12	0.17	0.09	-0.11	0.12	0.29	0.39	0.09	
6	1953.70	0.02	-0.12	0.16	0.09	-0.11	0.12	0.28	0.37	0.09	
7	1953.87	0.01	-0.17	0.16		-0.11	0.19	0.34	0.40	0.18	0.17
8	1954.03	0.01	-0.15	0.16	0.09		0.15	0.29	0.38	0.18	
9	1954.09	0.01	-0.19	0.16			0.22	0.34	0.40		0.17
10	1954.31	0.01	-0.08	0.16	0.11	-0.14		0.29	0.39	0.09	

### Model based predictions and validation

**Fig 3:**
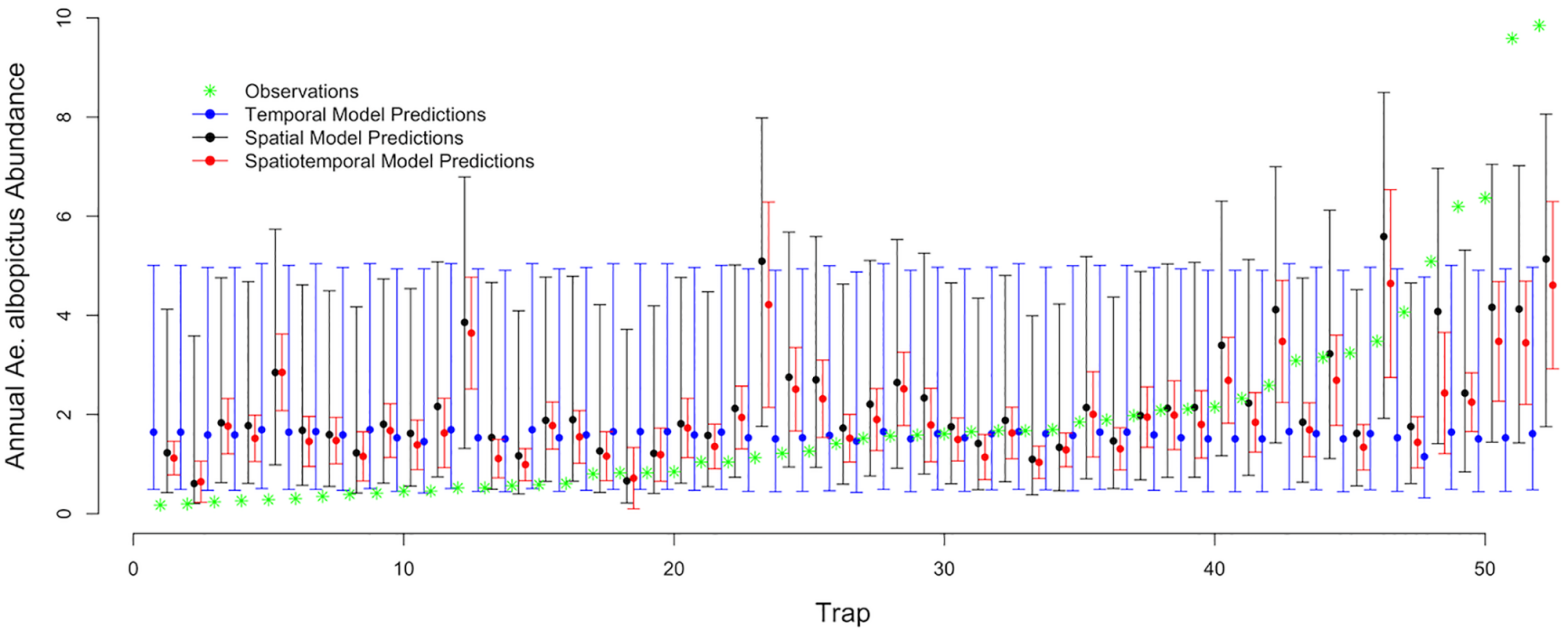
Comparison of model predictions. Model predictions for 2016 with 95% confidence intervals for the temporal model using meteorological conditions (blue), spatial model using local environmental conditions (black), and spatiotemporal model using a subset of important parameters from temporal and spatial modeling efforts (red). Green asterisks indicate actual 2016 observed *Ae*. *albopictus* abundance. On the x-axis, trap refers to the 52 permanent trap locations across NYC.

The temporal ensemble model predictions (made using monthly mean estimates of meteorological conditions) shows broad confidence intervals that are similar across all 52 permanent trap locations (Fig 3). This near uniformity is due to the small differences in meteorological conditions within NYC (S3 Fig). We used root mean squared error (RMSE) to compare the accuracy of the temporal, spatial, and spatiotemporal model predictions with the observed values for 2016. RMSE is largest for temporal ensemble predictions (2.58), followed by spatial ensemble predictions (2.25), and lowest for spatiotemporal ensemble predictions (1.75). RMSE for the LOOTCV model spanning all 11 years of analysis (RMSE = 2.38) and the full spatiotemporal model (RMSE = 2.34) predictions were comparable (S4 Fig), indicating that out-of-sample prediction is possible and that no single year overly dominates the model structure. Further we test the sensitivity and specificity of the spatiotemporal ensemble model predictions. We use the mean value of both the observed and predicted values (2.37) as the cut-off point for the analysis. we find that the sensitivity (to truly predict above average observed values) is 69% and the specificity (to truly detect below average observed values) is 77%.

### Risk of autochthonous disease spread

**Fig 4:**
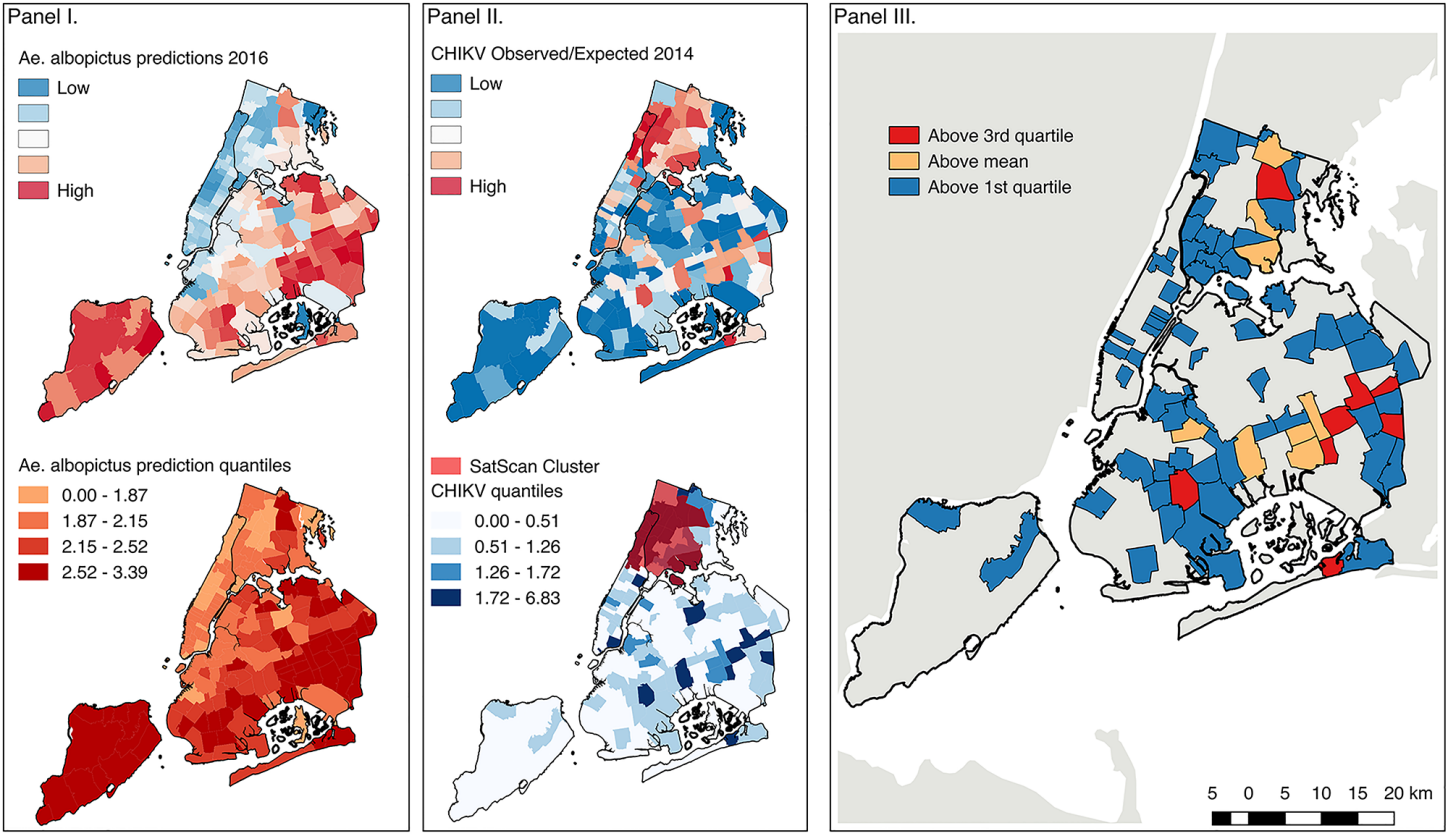
Risk map. Ensemble spatiotemporal model predictions of *Ae*. *albopictus* annual abundance for 2016 averaged by zipcode (top) and classified into quantiles (bottom). Panel II: Spatial distribution of imported CHIKV cases by zipcode in 2014 (top) and classified into quantiles with SatScan Cluster of CHIKV cases (bottom). Panel III: Spatial risk map combining data from the predicted mean value of *Ae*. *albopictus* abundance in 2016 with imported CHIKV cases from 2014 by zipcode. (Data sources: Entomological and Epidemiological data from the NYC DOHMH; meteorological data from NLDAS; environmental data from 3 foot landcover dataset (University of Vermont Spatial Analysis Laboratory and NYC Urban Field Station) and PLUTO; and the underlying geographic boundaries from 2014 TIGER/Line Shapefiles prepared by the U.S. Census Bureau).

To map predicted *Ae*. *albopictus* abundance for 2016 across NYC at fine spatial resolution we used the ensemble coefficient estimates from the spatiotemporal modeling effort and the surface raster grids created for each parameter (Fig 4, Panel I; S5 Fig). *Ae*. *albopictus* are predicted to be most abundant in parts of Staten Island, and southern Brooklyn and Queens.

During 2014 both imported CHIKV cases and *Ae*. *albopictus* abundance peaked in August suggesting that epidemic risk coincided temporally with mosquito abundance. In Fig 4 (Panel II) the spatial distribution of imported CHIKV cases is presented by zipcode. Zipcodes with higher risk are in northern Manhattan and the Bronx. Overlaid are the results from the spatiotemporal Poisson probability model run in SatScan (Fig 4, Panel II, bottom). Through this analysis we find a significant cluster of imported CHIKV cases between the months of July and October across 28 zipcodes verifying increased risk in upper Manhattan and the Bronx.

Using the mean predicted values of *Ae*. *albopictus* annual abundance by zipcode in conjunction with the distribution of imported CHIKV cases from 2014 we are able to ascribe risk for local transmission in NYC. We find that the distribution of imported CHIKV cases and areas of high *Ae*. *albopictus* abundance are mainly discordant; however, there are some areas of concordance, including parts of southern Queens in the vicinity of John F. Kennedy airport, as well as the Bronx (Fig 4; Panel III). These delineated areas of higher risk should inform vector control and public health personnel where to target control for *Ae*. *albopictus*-borne disease.

## Discussion

Here, we examined the separate temporal and spatial influences, as well as the combined spatiotemporal influences, on annual *Ae*. *albopictus* abundance in NYC using ensemble modeling methods. We find that spatial variability is greater than temporal variability, suggesting that local environmental conditions are a stronger determinant of *Ae*. *albopictus* abundance than inter-annual differences in meteorological conditions. This may be due to a general availability of hospitable meteorological conditions in NYC (S3 Fig) or may reflect the finer spatial resolution of the local environmental conditions compared to that of the meteorological data used in the analysis. Taken at face value, this finding underscores a greater importance of local environmental predictors over meteorological effects on annual *Ae*. *albopictus* abundance. However the improvement of model fit with the inclusion of both meteorological and environmental conditions indicates the importance of both for predicting annual *Ae*. *albopictus* abundance.

Meteorological conditions in the spring and early summer (February specific humidity, April and June precipitation, and June temperature) positively influence *Ae*. *albopictus* abundance. Higher February specific humidity indicates wetter, warmer conditions in February—conditions that may improve survivorship of overwintering eggs. April and June precipitation may increase container habitat for *Ae*. *albopictus*, leading to an increase in overall annual *Ae*. *albopictus* abundance. The influence of early season rainfall may be because rainfall early in the season is more directly linked to *Ae*. *albopictus* production than rainfall later in the breeding season which is decoupled from mosquito production by human watering activities [51]. Warmer temperatures in early summer, i.e. June, may lead to an acceleration of *Ae*. *albopictus* reproduction early in the season which may in turn lead to higher annual numbers. The importance of early season meteorological conditions suggests that annual predictions can be made before *Ae*. *albopictus* populations peak in NYC, which may help vector control initiatives target and reduce these pestiferous mosquitoes.

Of the environmental parameters tested we find that open spaces, residential areas, vacant lots, water, and grass influence *Ae*. *albopictus* abundance. The land use classifications (residential, open spaces, and vacant lots) were more important than individual land cover categories or the SDI in predicting *Ae*. *albopictus* abundance. Land use classifications depict a particular configuration of land cover types. Within open spaces, mainly parks in NYC [39] we find 35% of the area is trees, 39% grass, and only 1% buildings. These areas had a negative influence on the annual abundance of *Ae*. *albopictus*. Vacant lots have a similar composition of trees (25%), grass (37%), and buildings (7%) as open spaces albeit with fewer trees and more buildings. In contrast to open spaces, vacant lots had a positive influence on annual *Ae*. *albopictus* abundance. This difference may be explained by how humans engage with these different land use classifications. Unlike open spaces, vacant lots tend to be unmanaged areas where weedy vegetation is left and trash accumulates; characteristics noted by others to be associated with higher *Ae*. *albopictus* infestation [52–55].

Residential areas in NYC have a more equitable distribution of trees (21%), grass (15%), and buildings (25%) and were the most important environmental parameter predicting high *Ae*. *albopictus* populations. Residential areas and vacant lots, likely have more available containers than open spaces; however, the types of containers may differ substantially between areas designated as residential or vacant lots; with more permanent water holding containers more closely linked with human watering in residential areas compared to more discarded water holding containers more closely linked with rainfall in vacant lots [56]. The positive influences of both residential and vacant areas on annual *Ae*. *albopictus* populations suggest that habitat requirements are met in these locations. Further, because these mosquitoes do not travel far during their lifetimes, this indicates that the habitat requirements of this vector are met at both immature and adult life stages. While water holding containers suitable for mosquito development are present in both environments, the types of containers likely differ substantially [54, 57]. Thus when it is dry *Ae*. *albopictus* populations may only flourish in residential areas and future analysis should investigate the interactive effects of meteorological conditions, in particular precipitation, with land use classifications to further examine the influence of sociological processes on *Ae*. *albopictus* populations.

The data used for this analysis are somewhat limited by the trap types and locations of collection. The 52 permanent trap locations were installed in 1999 after the introduction of WNV in NYC and both the trap locations and trap types deployed are specifically targeted to collect WNV vectors (i.e. *Culex* mosquitoes). The trap types, light and gravid traps are not as well suited to capture *Ae*. *albopictus* compared to other traps such as BG Sentinel traps. BG Sentinel traps were deployed for the first time in 2016, and the results of this analysis can be used to further inform placement of BG Sentinel traps to areas predicted to have high *Ae*. *albopictus* populations. 70% (n = 37) of the permanent trap locations are within park boundaries in NYC. While other researchers have found that small green islands within urban areas are hot spots for *Ae*. *albopictus* and disease transmission [26, 58, 59], the results of this analysis suggest that residential areas are likely to have higher *Ae*. *albopictus* populations than park land in NYC. Thus, while the current surveillance provides an important time series of annual *Ae*. *albopictus* abundance, an expansion of trap locations to reflect local environmental conditions that favor *Ae*. *albopictus* such as in residential areas and vacant lots may provide better population estimates.

While the spatiotemporal ensemble model predictions for 2016 capture the range of observations, they overestimate annual *Ae*. *albopictus* abundance when observations are low and underestimate annual *Ae*. *albopictus* abundance when observations are high (Fig 3). This limitation may be due to the spatial or temporal scales on which we based our measurements. Indeed, the predictive capability of the spatiotemporal model may be improved by incorporating measures of meteorological and environmental conditions at different scales. However, the sensitivity and specificity tests support the ability of the model to distinguish between above and below average years of *Ae*. *albopictus* production which is important for informing vector control initiatives.

In evaluating the risk of local arbovirus transmission, we find that the distribution of *Ae*. *albopictus* and imported CHIKV cases is temporally aligned (Fig 4, Panel II, bottom) but primarily spatially discordant, which provides a check on local transmission in NYC. However, we do identify locales at higher risk (Fig 4, Panel III), which should provide guidance for future vector surveillance and control as well as public health educational campaigns. The distribution of imported DENV and ZIKV cases should be compared to the CHIKV cases mapped here to determine any similarities or differences in the distribution of imported arboviruses across NYC and assess if the spatiotemporal distribution of imported CHIKV case is suitable for ascribing overall risk of arboviral introduction into local *Ae*. *albopictus* populations.

Local transmission of CHIKV by *Ae*. *albopictus* has not been reported in NYC likely due to a combination of the strain currently circulating in the western hemisphere and socioeconomic conditions in the northeastern US that limit vector-host contact rates. The CHIKV strain circulating in the western hemisphere belongs to the Asian lineage, while local CHIKV transmission by *Ae*. *albopictus* in temperate Europe is linked to the CHIKV variant (E1—226V) which is more readily transmitted by *Ae*. *albopictus* [60–62]. A future introduction of the E1—226V variant might thus lead to local CHIKV transmission in the northeastern US by *Ae*. *albopictus*.

In temperate areas, *Ae*. *albopictus* is the only endemic vector of CHIKV as well as DENV and ZIKV. Its broad viral susceptibility suggests that it may be implicated as an important, if not primary, vector in the transmission of other arboviruses now and in the future [11]. Blood titers from imported human cases have documented levels sufficient to infect endemic mosquito vectors [63]. Therefore, the introduction of just one case could trigger a local outbreak [21], especially if vector densities are high [64, 65]. In the northeastern US, because human population density and susceptibility are high and the population is unfamiliar with protective behaviors, arboviruses could spread quickly [1].

Socioeconomic factors, in particular, window screens and access to air conditioning (AC) that limit vector-host contact rates, have restricted the temperate spread of mosquito borne disease in the US [66, 67]. While these barriers are typically sufficient against vector borne diseases in the US, their distribution remains inequitable and their permanence is not guaranteed. In NYC access to AC is variable, with up to 40% of senior citizens in areas of Brooklyn and the Bronx reporting no access [68]. Further analysis could incorporate social risks such as these to better focus vector control and public health education efforts. Additionally, climate-related extreme weather events are expected to produce increased damage to infrastructure and power outages, which could significantly alter mosquito-human contact rates.

### Conclusion

*Ae*. *albopictus* is a pestiferous mosquito that reduces outdoor use and effectively transmits a number of emergent arboviruses [4]. Currently there are no vaccines or treatments available for these arboviruses. Limiting disease transmission still hinges on effective vector control, which depends on removal and/or regular maintenance of containers, efforts that require concerted, coordinated efforts between vector control officers and communities. Entomological surveillance records widespread and abundant *Ae*. *albopictus* populations in NYC (Table 1; Fig 1) despite ongoing vector control efforts. Because these mosquitoes are so difficult to control informed, targeted vector control efforts are essential. To this end, we have identified key meteorological and local environmental conditions associated with *Ae*. *albopictus* abundance, developed spatiotemporal models of *Ae*. *albopictus*, and generated spatially explicit forecasts of this risk in NYC. By overlaying the spatiotemporal ensemble model of *Ae*. *albopictus* abundance with potential arbovirus introduction risk as determined by the spatiotemporal distribution of imported CHIKV cases in 2014, we delineate fine scale spatial differences in local arbovirus transmission risk in NYC that may be used to guide vector control and public health educational campaigns.

### Supporting information

#### Environmental and meteorological conditions

Temperature and precipitation are two meteorological conditions that are known to directly impact *Ae*. *albopictus* populations. Temperature can have both direct and indirect influences on adult and juvenile survival, juvenile development, and adult female biting behaviors [69–73]. Optimal temperatures for *Ae*. *albopictus* survival lie between 25 and 30C with mortality under 15C and over 35C [51], though *Ae*. *albopictus* can lay diapausing eggs that survive subfreezing conditions. Likewise, precipitation is necessary to fill container habitats and maintain the water resources necessary for juvenile mosquito development [69, 74, 75]. The effect of precipitation is complicated by human activities, particularly watering in residential areas [56]. It is thought that spring precipitation directly increases container habitat but that later in the year, container conditions are decoupled from precipitation due to human watering [51]. We also examine specific humidity: when specific humidity is high temperature and rainfall measures are also high—conditions that are conducive to mosquito development, dispersal and survival of adult mosquitoes, and (if present) rapid replication of arboviruses [76, 77].

The meteorological data for NYC indicate that temperatures do not exceed the upper thresholds of survival (S3 Fig). Mortality for diapausing eggs is thought to occur when average January temperatures fall below −2C [78] which may constrain population growth the following breeding season. Between 2006 and 2016 mean January temperatures dropped below −2C in 2009, 2011, 2014, and 2015—although not at all trap locations.

Empirical models from the northeastern US suggest that local environmental conditions, including land cover, as well as human behaviors, underlie spatial differences in *Ae*. *albopictus* populations. *Ae*. *albopictus* populations have been linked to human population density [15, 63, 79], especially shaded residential areas [59, 79, 80]. Residential areas may provide the highest frequencies of suitable artificial containers [55, 75, 79]; however, residential areas with diffuse tree canopy may provide the best habitat conditions for *Ae*. *albopictus*. Trees are the most important contributor of food resources to developing immatures [54, 79, 81, 82]; trees also provide shade that reduces evapotranspiration (resulting in water remaining in containers longer increasing the likelihood of successful immature development) [83, 84]; and provide shaded environments for adult resting [57]. In the Northeastern US, *Ae*. *albopictus* populations are further linked to socioeconomic status; with lower socioeconomic neighborhoods experiencing higher *Ae*. *albopictus* infestation linked to higher amounts of disused containers [52, 53, 85].

For many of the environmental parameters, the conditions around the trap locations do not represent the full range of conditions found in NYC (S6 Fig). This is especially true for bare surfaces, roads, diversity, water, and vacant lots and may have implications for predicting annual *Ae*. *albopictus* in areas with environmental conditions not represented by the trap locations.

## Supporting information

S1 Fig 52 permanent trap locations. Stars represent location of the 52 permanent trap locations operated across NYC during the study period.
(TIFF)

S2 Fig Methods schematic. A visual representation of the methodological steps taken. Temporal and Spatial modeling conducted separately using ensemble modeling methods. Parameters of high importance were employed in a unified spatiotemporal ensemble modeling approach to reach the final ensemble model used to make predictions.
(TIF)

S3 Fig Meteorological conditions. Variability of meteorological conditions across 11 years of observations (2006–2016) across all trap locations in NYC.
(TIF)

S4 Fig Temporal cross validation. Full model (all years) predictions and observations (left panel) compared to temporal cross validation predictions and observations (right panel).
(TIF)

S5 Fig Spatiotemporal model predictions. Ensemble Spatiotemporal model predictions of *Ae*. *albopictus* for 2016. (Data sources: Entomological and Epidemiological data from the NYC DOHMH; meteorological data from NLDAS; environmental data from 3 foot landcover dataset (University of Vermont Spatial Analysis Laboratory and NYC Urban Field Station) and PLUTO; and the underlying geographic boundaries from 2014 TIGER/Line Shapefiles prepared by the U.S. Census Bureau).
(TIFF)

S6 Fig Environmental conditions. Variability of each environmental parameter across trap locations. The red boxes indicate full extent of variability of environmental parameters across full domain of NYC.
(TIF)
