# Supplementary figures and images for: Local environmental and meteorological conditions influencing the invasive mosquito Ae. albopictus and arbovirus transmission risk in New York City
Source: PLoS Negl Trop Dis. 2017 Aug 23;11(8):e0005828. doi: 10.1371/journal.pntd.0005828 (PMC5584979; doi:10.1371/journal.pntd.0005828)

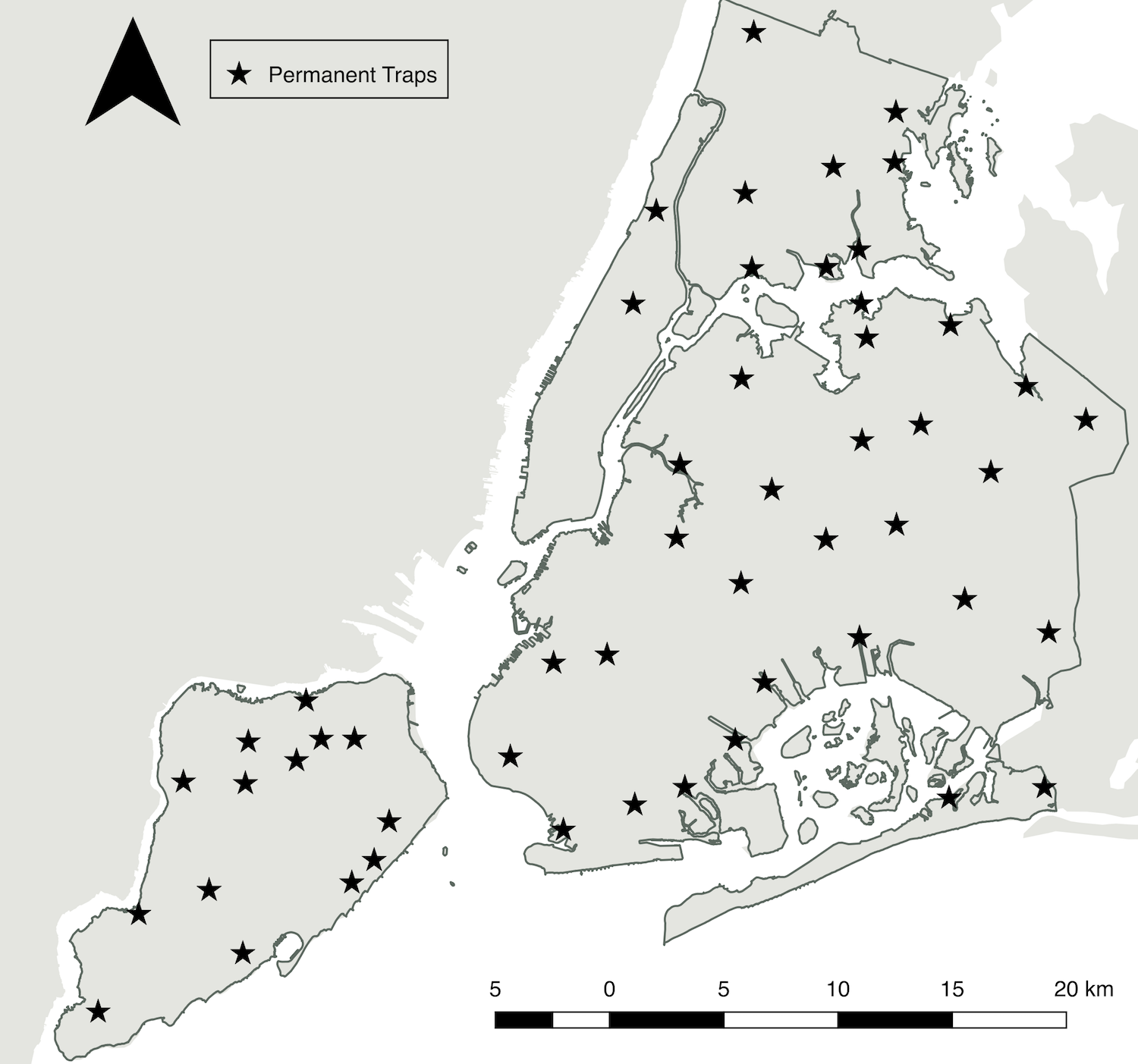

Supplement: S1 Fig — Stars represent location of the 52 permanent trap locations operated across NYC during the study period. (TIFF) [file pntd.0005828.s001.tiff]

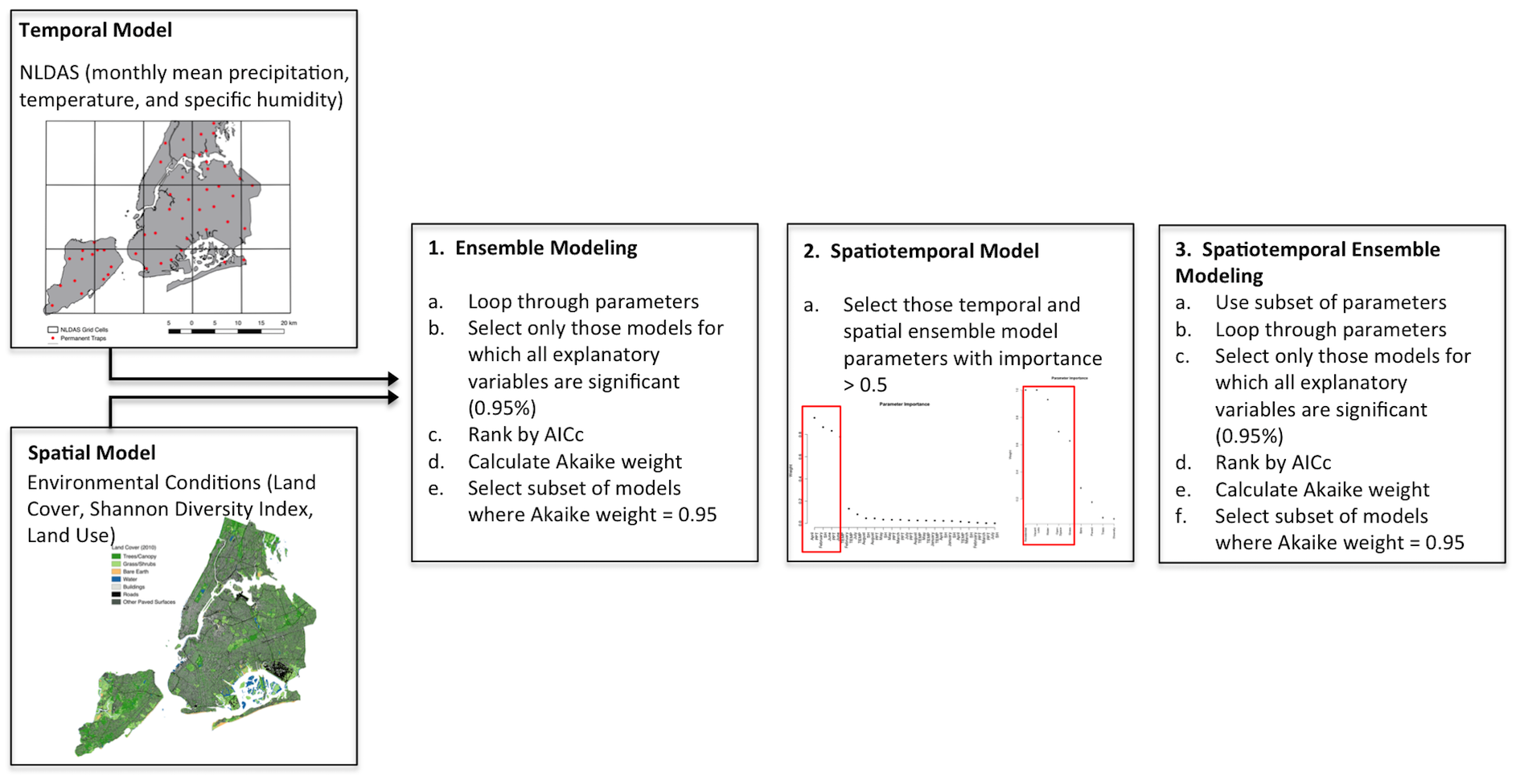

Supplement: S2 Fig — A visual representation of the methodological steps taken. Temporal and Spatial modeling conducted separately using ensemble modeling methods. Parameters of high importance were employed in a unified spatiotemporal ensemble modeling approach to reach the final ensemble model used to make predictions. (TIF) [file pntd.0005828.s002.tif]

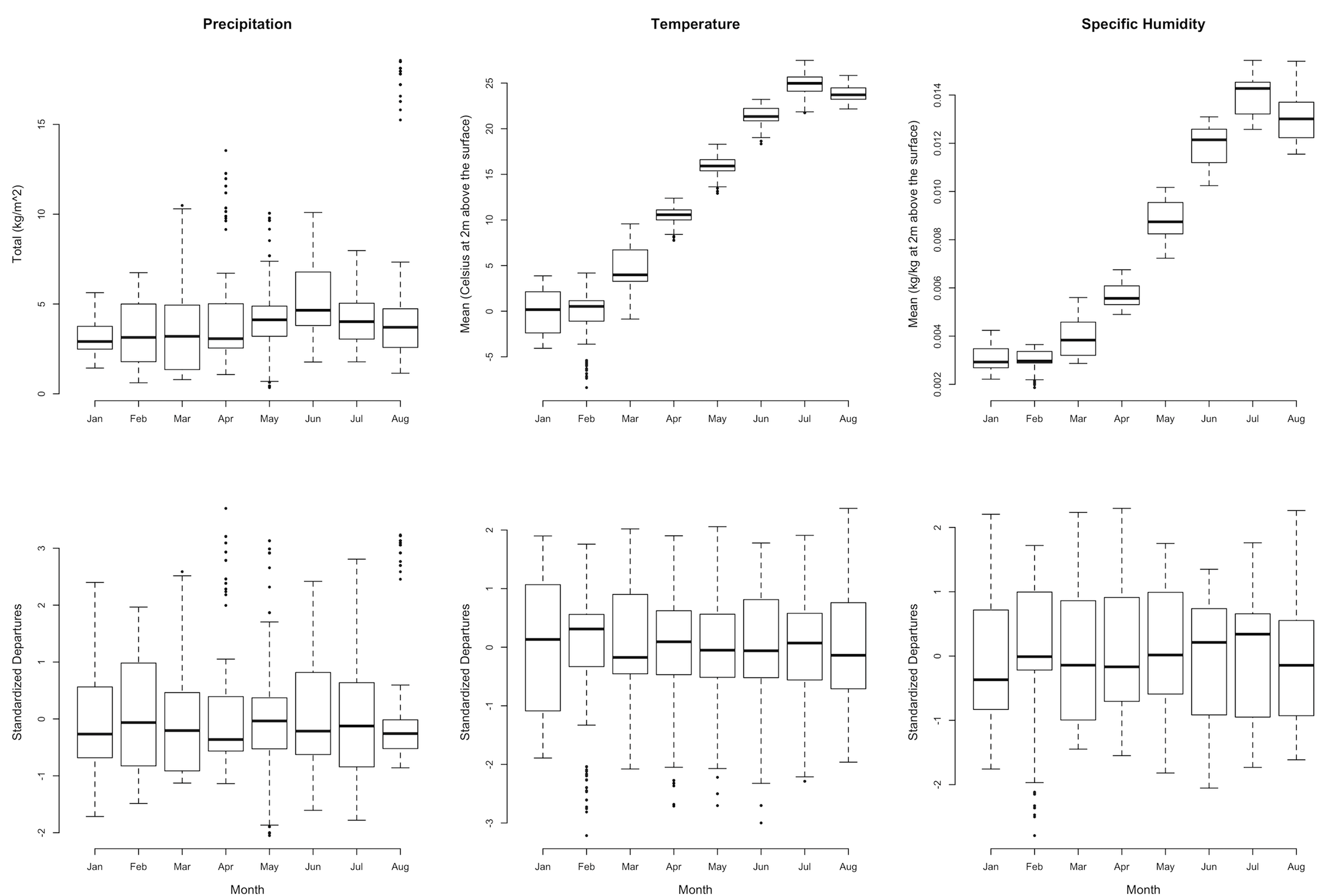

Supplement: S3 Fig — Variability of meteorological conditions across 11 years of observations (2006–2016) across all trap locations in NYC. (TIF) [file pntd.0005828.s003.tif]

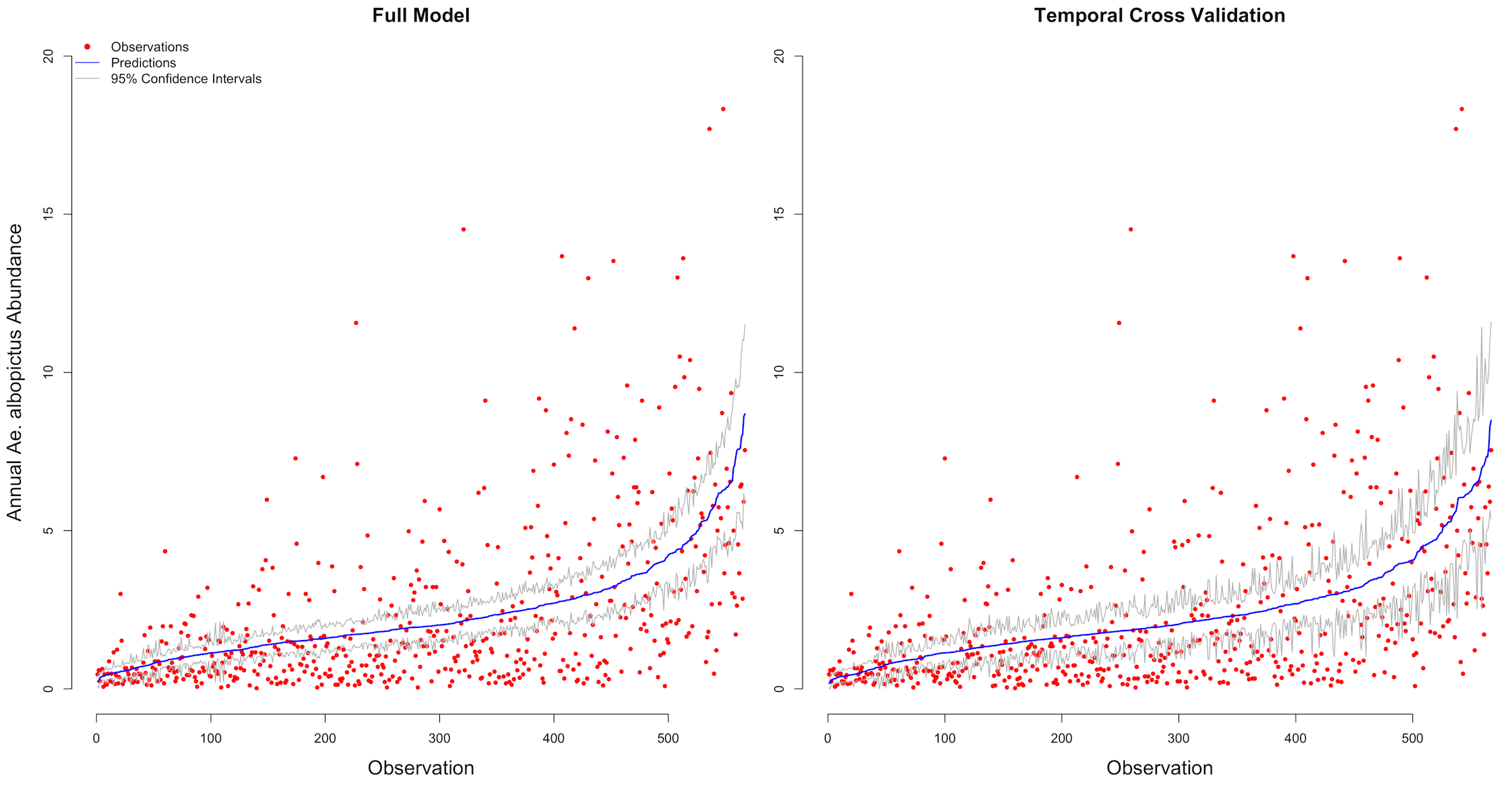

Supplement: S4 Fig — Full model (all years) predictions and observations (left panel) compared to temporal cross validation predictions and observations (right panel). (TIF) [file pntd.0005828.s004.tif]

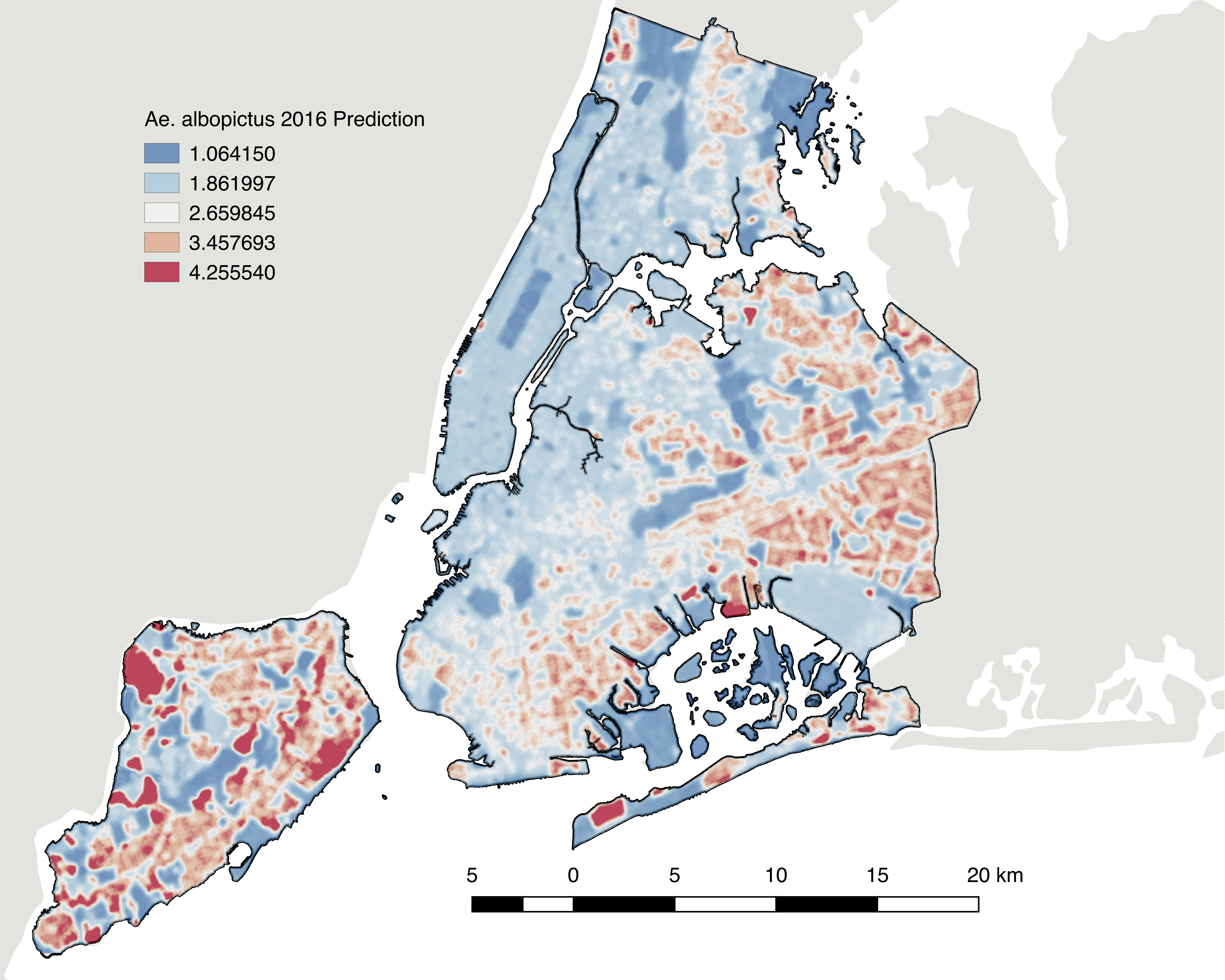

Supplement: S5 Fig — Ensemble Spatiotemporal model predictions of Ae. albopictus for 2016. (Data sources: Entomological and Epidemiological data from the NYC DOHMH; meteorological data from NLDAS; environmental data from 3 foot landcover dataset (University of Vermont Spatial Analysis Laboratory and NYC Urban Field Station) and PLUTO; and the underlying geographic boundaries from 2014 TIGER/Line Shapefiles prepared by the U.S. Census Bureau). (TIFF) [file pntd.0005828.s005.tiff]

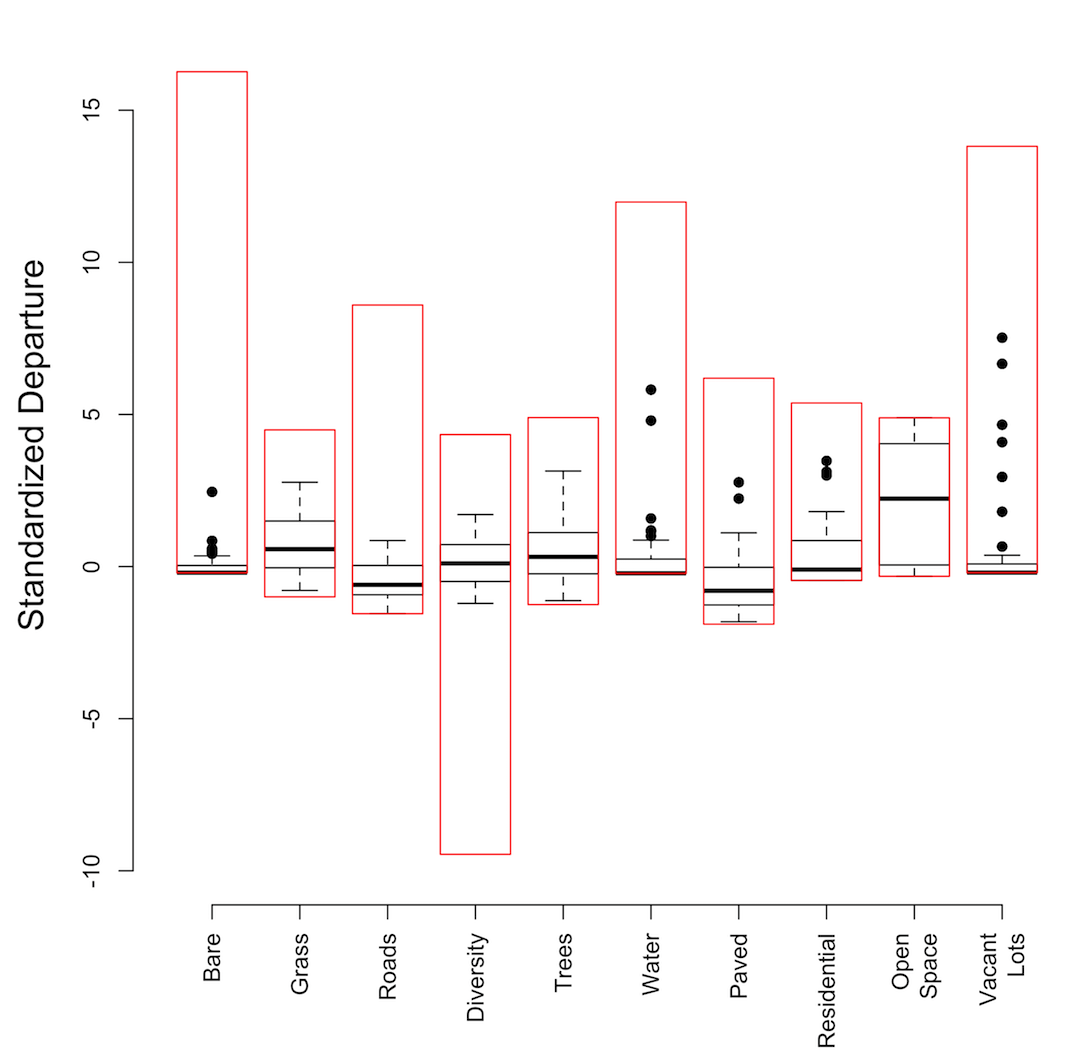

Supplement: S6 Fig — Variability of each environmental parameter across trap locations. The red boxes indicate full extent of variability of environmental parameters across full domain of NYC. (TIF) [file pntd.0005828.s006.tif]
